# The Defibrillation Conundrum: New Insights into the Mechanisms of Shock-Related Myocardial Injury Sustained from a Life-Saving Therapy

**DOI:** 10.3390/ijms22095003

**Published:** 2021-05-08

**Authors:** Nicolas Clementy, Alexandre Bodin, Arnaud Bisson, Ana-Paula Teixeira-Gomes, Sebastien Roger, Denis Angoulvant, Valérie Labas, Dominique Babuty

**Affiliations:** 1 Service de Cardiologie, Hôpital Trousseau, Université de Tours, 37044 Tours, France; alexandrebodin.mail@gmail.com (A.B.); arnaud.bisson37@gmail.com (A.B.); denis.angoulvant@univ-tours.fr (D.A.); d.babuty@chu-tours.fr (D.B.); 2 Transplantation, Immunologie et Inflammation T2I-EA 4245, Université de Tours, 37044 Tours, France; sebastien.roger@univ-tours.fr; 3 Plate-forme de Chirurgie et d’Imagerie pour la Recherche et l’Enseignement (CIRE), INRA, Université de Tours, CHU de Tours, 37380 Nouzilly, France; ana-paula.teixeira@inrae.fr (A.-P.T.-G.); valerie.labas@inrae.fr (V.L.)

**Keywords:** defibrillation, mechanism, injury, electrical, shock, proteomics

## Abstract

Implantable cardiac defibrillators (ICDs) are recommended to prevent the risk of sudden cardiac death. However, shocks are associated with an increased mortality with a dose response effect, and a strategy of reducing electrical therapy burden improves the prognosis of implanted patients. We review the mechanisms of defibrillation and its consequences, including cell damage, metabolic remodeling, calcium metabolism anomalies, and inflammatory and pro-fibrotic remodeling. Electrical shocks do save lives, but also promote myocardial stunning, heart failure, and pro-arrhythmic effects as seen in electrical storms. Limiting unnecessary implantations and therapies and proposing new methods of defibrillation in the future are recommended.

## 1. Introduction

Implantable cardiac defibrillators (ICDs) are recommended to prevent the risk of sudden cardiac death. More than 300,000 ICDs worldwide are implanted each year. However, shocks are associated with an increased mortality with a dose response effect, and a strategy of reducing electrical therapy burden improves the prognosis of implanted patients. We review the mechanisms of defibrillation and the potential consequences of ICD shocks, including cell damage, metabolic remodeling, calcium metabolism dysregulation, inflammation, and fibrosis.

## 2. Background

### 2.1. Technology

**Figure 1:**
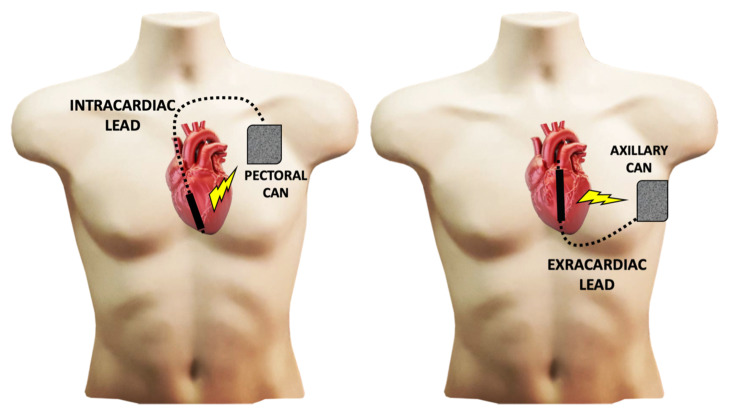
Main implantable cardiac defibrillators (ICD) technologies on the market. The historical technique uses a subcutaneous or submuscular can and an intracardiac lead implanted in the right ventricle through supra-caval venous access (Left Panel). Extracardiac technologies use an axillary can and a defibrillation lead tunneled subcutaneously in a para-sternal position or directly under the sternum in a para-cardiac position (Right Panel). Shocks are delivered between the hot can and the defibrillation lead coil.

Defibrillation has come a long way since 1775, when a Danish veterinarian, Peter Christian Abildgaard, wishing to understand the destructive effects of lightning on animals, using Leyden bottles (the ancestor of the capacitor) on hens, noticed that a shock to the chest could resuscitate them [1,2]. The first ICD model was a simple shock box designed by Mieczyslaw Mirowski [3]. The device, which weighs 289 g or 150 mL, is implanted into the abdominal area and requires a sternotomy to place two epicardial patches for defibrillation and two screwed leads for detection. Technical improvements have never ceased: the synchronized electric shock was developed in 1982, which allows the reduction of monomorphic ventricular tachycardias; a combined system including a subcutaneous patch and an endocardial ventricular lead with coil was developed in 1988, removing the need for thoracotomy; a biphasic shock was developed in 1991; an exclusively transvenous system was developed in 1995 with a box in the pectoral position; and anti-tachycardia pacing was developed in 1996. From the 2000s onwards, all ICDs have had anti-bradycardia pacing capabilities as sophisticated as those of pacemakers, since the arrival of biventricular resynchronization in 2003, complex rhythm detection algorithms, discrimination of rhythms of ventricular or supra-ventricular origin, and defibrillation with an active box (hot can) have become possible. A totally extra-cardiac device, the subcutaneous ICD, emerged a decade ago [4] (Figure 1).

### 2.2. Indications

#### 2.2.1. Secondary Prevention

ICDs are used to treat patients who have suffered a sudden death, or in any case a sustained arrhythmia of ventricular origin. It seems incredible today to think that the ICD was used at the beginning not in case of sudden death, but in case of recurrence of sudden death! Only the most miraculous benefited from it. It was not until the end of the 1990s that the results of the first large randomized studies became available [5,6,7]. In AVID, which involved more than 1000 patients who had recovered from ventricular fibrillation or ventricular tachycardia and left ventricular systolic dysfunction, the ICD reduced total mortality at 1 year by 39% compared with anti-arrhythmic treatment with amiodarone (10.7% versus 17.7% in absolute risk).

ICDs are now recommended (class I, level of evidence B) in patients with ischemic heart disease, who either survive sudden cardiac arrest due to ventricular arrhythmia or experience hemodynamically unstable or stable sustained ventricular tachycardia due to irreversible causes [8].

#### 2.2.2. Primary Prevention

Primary prevention involves the prophylactic treatment of patients at high risk of ventricular arrhythmia or sudden death. The vast majority of these patients have heart disease with severe left ventricular systolic dysfunction complicated by heart failure. The MADIT study opened the way in 1996 for post-infarction patients with inducible ventricular arrhythmia, with a 54% reduction in total mortality in favor of the ICD [9]. MUSTT confirmed these results in 1999 in more than 700 patients [10]. The large post-infarction study, MADIT II, was published in 2002 [11]. A total of 1232 patients with a left ventricular ejection fraction (LVEF) less than or equal to 30% were randomized: the ICD significantly reduced mortality by 31% after 20 months of average follow-up. The SCD-HeFT study in 2005 confirmed the benefits of ICDs in patients with non-ischemic heart disease and heart failure, although these results were more recently qualified by the negative results of the DANISH study [12,13].

ICDs are now recommended (class I, level of evidence A) in patients with non-ischemic cardiomyopathy, and in ischemic patients at least 40 days after myocardial infarction, and at least 90 days post revascularization in case of heart failure (HF) with NYHA class II or III and an LVEF of 35% or less [8].

### 2.3. Less Is More

A distinction is usually made between appropriate electric shocks, which are delivered due to an arrhythmia of ventricular origin, and inappropriate shocks, which are delivered due to a supra-ventricular arrhythmia (mostly atrial fibrillation) or noise oversensing (electromagnetic interference, myopotentials, defibrillation lead fracture, T-wave oversensing, etc.). In reality, the line between appropriate and inappropriate is much more blurred. There are also treatments that are likely unnecessary or superfluous, making them very difficult to quantify.

If ventricular fibrillation rarely stops spontaneously, ventricular tachycardias, on the contrary, are mostly non-sustained (less than 30 s, by definition). This has been well demonstrated in studies that have compared a short detection time before delivery of therapy with a longer detection time. In ischemic heart disease, the PREPARE study found a 61% reduction in inappropriate shocks and a 45% reduction in appropriate shocks when the detection time was increased to 30 of 40 intervals [14]. In RELEVANT, which focused on non-ischemic dilated heart disease, changing the detection time from 12/16 to 30/40 intervals, which represents only 5 s at a rate of 200 bpm, withheld 91% of treatments [15]! It is important to note that these studies did not report any deleterious effect of programming slower treatments, either on symptoms (syncope) or morbidity.

Substudies of large randomized trials show that shocks are independently associated with a poor prognosis. For example, in MADIT II, hazard ratios for all-cause mortality were 3.4 for appropriate shock, 2.3 for inappropriate shock, and more than 4 for any shock [16]. In SCD-HeFT, the hazard ratios were even higher, at 5.7, 2.0, and more than 11, respectively [17].

The logical assumption would be that patients with the most severe forms of heart disease have the most ventricular arrhythmias. Appropriate shocks will therefore intervene mainly in patients with the worst prognosis. Sweeney has thus shown in a large retrospective of 4 randomized trials that patients who have the most therapies through their ICD have the most recurrences of ventricular arrhythmias, and the worst prognosis, with a 20% excess risk of death per additional shock [18]. It would also be logical to conclude that patients who have inappropriate shocks are mostly those who have episodes of rapid atrial fibrillation (by far the greatest source of inappropriate therapy), and who therefore have a worse prognosis, since the presence of atrial fibrillation may worsen heart failure [19].

Or could there be a deleterious effect (injury) specific to ICD shocks themselves, independent of the underlying cardiac disease?

### 2.4. Limiting Unnecessary Implantable Cardiac Defibrillators (ICD) Shocks

Implanting a defibrillator is above all preventing an event that is rare (sudden death). The ICD is not a treatment for ventricular arrhythmias, unlike anti-arrhythmic drugs or radiofrequency ablation, in the sense that it does not modify the arrhythmogenic substrate.

In case of recurrent ventricular arrhythmia, it is recommended, especially in patients with ischemic heart disease and monomorphic ventricular tachycardia, to optimize antiarrhythmic medical therapy with a beta blocker, amiodarone, or sotalol [8]. Optimization of HF therapy is also recommended. Catheter ablation of the ventricular scar has also proven to dramatically reduce ventricular arrhythmia recurrence and improve patient outcomes [20,21].

Minimalist programming was the origin of the Very-High-Rate Registry, which prospectively includes more than 500 patients implanted with an ICD in primary prevention, all programmed with a single high-rate therapy zone above 220 beats-per-minute [22]. The incidence of appropriate (3.5% per year) and inappropriate (2% per year) therapy is extremely low in patients with structural heart disease and severe left ventricular systolic dysfunction. Adverse events are also very rare: only 1.9% of symptomatic patients have ventricular arrhythmias between 170 and 220 bpm (untreated), and no deaths have been attributed to programming.

Our preliminary study was followed by the MADIT-RIT trial, which brought everyone into alignment [23]. A total of 1500 patients implanted with an ICD in primary prevention for heart disease with left ventricular systolic dysfunction (53% ischemic) were randomized into 3 groups: “standard” programming with rapid therapies starting at 170 bpm, “high-frequency” programming with therapies only beyond 200 bpm, and finally “long delay” programming with extended detection times. The results were spectacular, with a reduction in the total number of therapies of 82% in the “high frequency” group and 68% in the “long delay” group compared to the “standard” group, but above all a reduction in overall mortality of 55% and 45%, respectively. A simple change in ICD programming to reduce the rate of therapy had a direct impact on the prognosis of implanted patients by halving their risk of death. In a substudy, after adjustment, appropriate shocks increased the risk of death by more than 6, while inappropriate therapies increased it by almost 3 [24].

MADIT-RIT is a landmark study. There seems to be a causality between therapies and morbidity and mortality. Whereas intuitively doctors tended to over-treat patients to protect them (but above all to reassure themselves), they must now avoid imposing on them as much as possible the deleterious effects of treatments, especially shocks [25]. In a controlled study published in 2017, we compared the results of the Very High-Rate Registry (VH-RATE programming with therapies only from 220 bpm in primary prevention) to other types of programming through the French national DAI-PP registry [26]. After adjustment by propensity score, compared to aggressive programming with two active therapy zones, VH-RATE programming reduced the risk of appropriate therapy by 62%, inappropriate therapy by 56%, and most notably sudden death by 57%. Compared with tolerant programming such as “MADIT-RIT high frequency”—a single zone of therapy above 200 bpm—mortality was similar, and the reduction in the risk of appropriate and inappropriate therapy was 50% and 53%, respectively.

Expert consensus guidelines on ICD programming were published to encourage physicians to change their habits, which are unfortunately persistent [27,28].

## 3. Potential Consequences of ICD Shocks

### 3.1. Sudden Death

The ICD prevents sudden death, not all sudden deaths. In SCD-HeFT, for example, deaths due to ventricular arrhythmia occurred in 11.2% of patients in the placebo group, 8.9% in the amiodarone group, and even 4.5% of patients in the ICD group, i.e., 1 in 5 deaths [29]. In a study of the cause of sudden death in 68 ICD patients, 29% were due to post-shock electro-mechanical dissociation (from the first shock in 40% of cases), and 25% were due to ineffective shocks [30]. These modes of death are particularly frequent in patients with severe, terminal, heart failure. In these patients, therapy, sometimes unnecessary, may precipitate death. Defibrillation testing is not recommended in this population.

### 3.2. Electrical Storm

Electrical shocks, often isolated, can also occur in a series: this is the electrical storm. Of course, direct causes can exist. The alternation between long and short cycles following defibrillation can re-initiate a ventricular arrhythmia. It is recommended to program a temporary post-shock rapid anti-bradycardia pacing to avoid this phenomenon. On the other hand, if detection is imperfect (over- or under-detection), a poorly synchronized electric shock delivered during the vulnerable period of repolarization may initiate ventricular fibrillation. These causes are rare, however, and it is likely that the arrhythmogenic effect of electric shocks is more often due to structural alterations in ventricular myocardial function.

An arrhythmic storm is usually defined as three or more episodes of ventricular arrhythmias (treated or untreated) within 24 h. It is associated with a very poor prognosis. In MADIT II, it involved 4% of patients, with 16% of patients having episodes of ventricular arrhythmias recorded by the ICD [31]. After adjustment, the risk of death within 3 months was increased 18-fold. In a more recent meta-analysis, the risk of death after electrical storms was increased by a factor of 3.2, and even by a factor of 2.5 compared with patients who had isolated episodes of ventricular arrhythmias without electrical storms [32]. Interestingly, again, as shown by the large Italian OBSERVO-ICD registry, a strategy of reducing electric shocks, by using ATP or increasing detection times and treatment rate threshold, was associated with a reduced risk of rhythmic storm, all-cause death, and death from heart failure [33].

### 3.3. Acute Heart Failure

Electrical storms are associated with an increased risk of hospitalization for acute HF and heart transplantation [32]. In MADIT II, patients with ICDs who received any therapy had an almost doubling of their risk of hospitalization for HF [34]. There is clearly a risk of post-shock myocardial stunning [35]. Early studies following programmed defibrillation threshold testing showed decreased cardiac output and alterations in diastolic function on echocardiographs [36,37,38]. More recently, a Japanese team has shown a significant reduction in left ventricular ejection fraction after defibrillation testing only in patients with prior left ventricular systolic dysfunction [39]. Systolic function remained impaired after 5 min and normalized after 4 h. This acute hemodynamic deleterious effect is also proportional to the energy delivered [40,41,42].

### 3.4. Adrenergic Storm

ICD therapies are known to impair quality of life in implanted patients, especially in the short-term [43]. Shock-induced stress and subsequent catecholamine release may even induce stress cardiomyopathy, also known as Takotsubo syndrome [44]. This adrenergic storm may thus favor myocardial stunning during the acute phase of ICD shocks, along with ventricular arrhythmia through positive bathmotropic effects.

**Figure 2:**
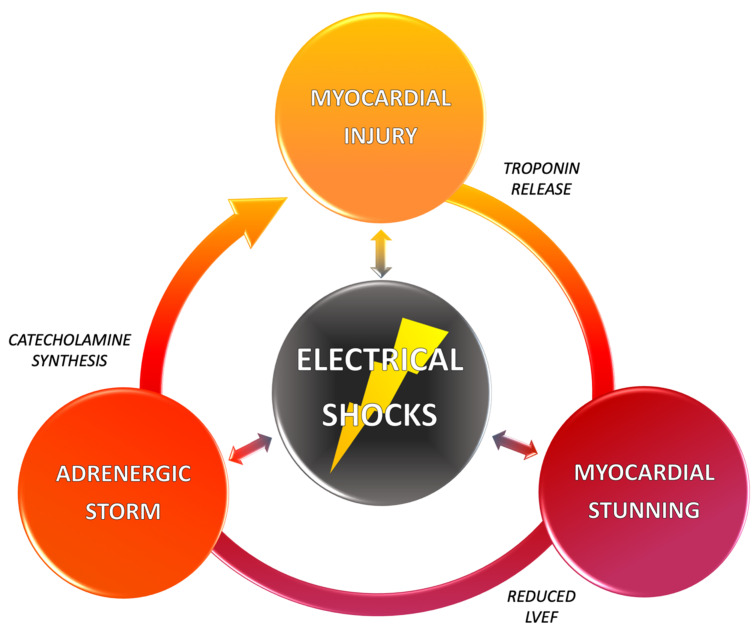
The vicious circle of ICD shocks. High-energy shocks induce myocardial injury, as shown by elevated troponin serum levels. They also induce myocardial stunning, and subsequent reduced left ventricular ejection fraction (LVEF) and cardiac output. They finally induce directly and through acute heart failure a major catecholamines release, with proarrhythmic effects, which increases the risk of arrhythmia reinduction and ICD shocks.

These studies undeniably show the deleterious role of shock by itself. By analogy with atrial fibrillation, “shocks beget shocks”. Shocks have an arrhythmogenic effect, favor arrhythmia recurrence, thus new therapies, and sometimes cause sudden death by electro-mechanical dissociation. They also favor the decompensation of chronic HF, often in the most vulnerable patients, which can precipitate death or cardiac transplantation (Figure 2).

## 4. Pathophysiology of Defibrillation

### 4.1. Mechanisms of Defibrillation

Although the delivery of an electrical current to the heart appears to be a fairly simple process, the mechanisms by which this current will lead to defibrillation are in fact extremely complex [45]. A direct electric current is delivered between 2 electrodes with a potential difference of about 800 V, for 5 to 10 milliseconds. The shape of the shock wave is biphasic in modern defibrillators: the direct current is delivered successively in one direction, then in the other. The current density is greatest in the immediate vicinity of the defibrillation electrodes, in contact with the right ventricular coil and the defibrillator case in a modern single-coil hot can configuration. According to Ohm’s law, at constant myocardial tissue resistivity, the current density is proportional to the potential gradient. Defibrillation is possible if this potential gradient across the entire ventricular myocardial tissue is greater than a critical value, about 4 V/cm for a standard biphasic shock (6 V/cm for a monophasic shock) [46].

However, it should be remembered that the defibrillation threshold is probabilistic. In modern ICDs, an optimally shaped biphasic shock has an ED90 (the energy required to obtain a 90% success rate) of roughly 15 J. It will therefore not always defibrillate. However, successive shocks of the same energy, in an identical configuration, produce the same potential gradient distribution [47]. The stochastic effect is therefore produced by the variations due to the unstable rhythm of ventricular fibrillation, with depolarization fronts and zones in the refractory period that are extremely variable at the time of the shock (mathematical theory of chaos) [48].

If the potential gradient is too low, a large zone, the furthest from the electrodes, will reactivate early and cause reinduction of the fibrillation by reentry phenomena [49]. Above this threshold, meanwhile, on the one hand the critical mass for re-initiation is not reached, and on the other hand the electric field leads to a prolongation of the duration of the action potentials initiated in the regions furthest from the electrodes. This is the graded response [50,51]. This prolongation of action potentials and refractory periods is proportional to the additional repolarization time caused by this graded response. It thus prevents the propagation of early post-shock activation fronts, thus decreasing the probability of fibrillatory wavelet reinduction. Thus, the success of defibrillation should rather be seen as the failure of post-shock ventricular reentry formation, which causes the failure of fibrillation reinduction [52].

At the cellular level, the effects of direct current have been well described by Weidmann [53]. In theory, during the first phase of a biphasic shock, it causes a hyperpolarization of the transmembrane potential in contact with the anode, and a depolarization in contact with the cathode, with an exponential decrease between the two depending on the distance to the electrode. Experimentally, due to the complexity of the distribution between extra- and intra-cellular media, and the orientation of the myocardial fibers (anisotropy), hyperpolarization is observed at the contact of the anode but depolarization at only a few millimeters, and the opposite at the cathode [54]. This virtual electrode effect results in a true patchwork of polarization, with the complex juxtaposition of depolarized and hyperpolarized regions [55]. And it is the smallest fascicular structures, with the largest surface curvature—the papillary muscles, for example—that are most sensitive to this polarization. The post-shock interaction of new depolarization fronts with these different regions of different polarization, refractory or not, allow or prevent the formation of perennial reentries, reinduction, or defibrillation. In the biphasic shock, the second phase, if well optimized in duration and voltage, allows homogenization of the myocardial tissue polarization between the two electrodes, erasing the virtual electrode patchwork and thus preventing reentry and facilitating defibrillation [56].

It is interesting to note that when the delivered energy is increased beyond a certain threshold, the probability of defibrillation actually decreases. Typically, above 1 kV and with gradient values above 150 V/cm, early ectopic activations appear in the regions where the gradient is highest, in contact with the electrodes, and facilitate the reinduction of ventricular fibrillation [57,58,59]. As the energy increases, the likelihood of bradyarrhythmia and post-shock tachyarrhythmia increases, especially when the shock is monophasic in form [60]. When the energy is too low, the reinduction of ventricular tachyarrhythmia is rather polymorphic, whereas it is rather monomorphic following a shock of excessive energy due to a local increase in the dispersion of repolarization [48,61].

Delivering a shock is therefore a matter of compromise: sufficient energy to have a high probability of defibrillation from the first shock (because in this early situation, the patient’s hemodynamics are not yet too compromised, and also to compensate for a possible increase in the resistance (impedance) of the defibrillating system over time); but a sufficiently low energy to avoid the reinduction of a post-shock ventricular arrhythmia.

### 4.2. Electroporation

When the lipid bilayer cell membrane is subjected to a strong electric field, its resistance decreases abruptly due to the formation of volcano-shaped, non-selective, aqueous pores permeable to ions and small molecules. They gradually expand to a diameter of 20–120 nm during the first 20 ms, then shrink and close after a few seconds [62]. This technique is commonly used for gene transfection.

Although the subject remains debated, this reversible electroporation could play a major role in defibrillation. Its beneficial effects would come from the fact that trabeculated endocardial structures are particularly sensitive—even at a distance—to polarization. Papillary muscles would thus be more susceptible to the effects of electroporation [63]. The Purkinje network, which is very involved in ventricular arrhythmogenesis, would therefore be more particularly affected by persistent post-shock conduction blocks, thus limiting the reinduction of ventricular fibrillation. Conversely, a prolonged state of membrane permeability results in potassium leakage into the extracellular medium, osmotic water flow, and especially intracellular calcium overload, thus favoring ventricular arrhythmias by afterdepolarizations.

When the electric field increases further, the electroporation becomes irreversible and leads to cell death. This technique is also used in medicine, for example in the treatment of tumors, or more recently proposed for the ablation of atrial fibrillation [64]. In myocardial tissue, the lethal dose 50 (to kill 50% of the cell population) corresponds to a potential gradient of about 80 V/cm for a biphasic electric shock [65]. This dose varies according to the orientation of the fibers, from 70 on average in the longitudinal direction to 140 V/cm in the transverse direction. Knowing that the maximum potential gradient of a shock at the defibrillation threshold, in contact with the electrode, is on average between 110 and 190 V/cm, depending on the configuration, one can imagine much higher values—and therefore larger lesions—on maximum energy shocks, even if the gradient decreases exponentially with distance [56]. In a histological study on the rabbit heart (Langendorff), a 300 V shock delivered to the tip of the right ventricle caused irreversible electroporation—measured by the intracellular penetration of a dye, propidium iodide—over about 200 mm^3^ opposite the electrode [66]. It was always transmural at the level of the free wall of the right ventricle (a little more than 2 mm thick), and a little less than 4 mm thick at the level of the interventricular septum. The extent of the lesions was comparable in healthy and infarcted hearts. This localized acute myocardial injury may play a role in the proarrhythmic and postinjury myocardial stunning effects.

### 4.3. Cellular Destruction

The first findings of release of myocardial necrosis markers after defibrillation are old [67]. A first study in humans on ICDs with epicardial patches did not find significant release of CPK-MB after defibrillation tests [68]. On the other hand, an increase in troponin I was observed by Joglar, with a peak in the first 12 h that was not explained by myocardial ischemia [69]. Hurst also found an elevation of troponin in 14% of the patients tested, reflecting discrete but real myocardial cellular destruction, particularly marked in those who had recently suffered an infarction [70].

This marker release occurs after programmed induction of ventricular fibrillation, and could therefore easily be attributed to the transient drop in cardiac output more than to the shock itself. However, a study of inappropriate electric shocks following a lead fracture, and therefore without underlying ventricular arrhythmia, found significant elevation of troponin in 3 out of 4 patients, and even very high levels—consistent with a medium-sized infarct or severe myocarditis—in 1 out of 10 patients [71]. These findings were confirmed in a larger German multicenter study, in which patients implanted with an ICD and tested, whether with or without induction of ventricular fibrillation, had a significant increase in ultra-sensitive troponin, in contrast to untested patients [72]. Finally, the large randomized SIMPLE study also found a significant elevation of troponin more frequent in the tested group [73].

The higher the energy delivered, the higher the damage appears to be [74]. The level of H-FABP (heart-type fatty acid binding protein), another very specific marker of necrosis in cardiac muscle, was higher after a high-energy shock. This elevated H-FABP level is associated with a worse long-term prognosis in ICD patients, with a higher rate of appropriate shocks and higher cardiovascular mortality [75]. Thus, electric shocks do cause measurable macroscopic cell death, and this could be a marker of poor prognosis.

After electric shock, cell death as a direct effect of the high-intensity electric field is evident, but it is probably not the only mechanism at work. A recent study by Brewster found not only an elevation of troponin in patients who had undergone a defibrillation test, but also a significant elevation of sFas, the soluble form of the transmembrane receptor Fas, which is involved in the amplification of the inflammatory response, the extrinsic pathway of apoptosis, and pro-fibrotic mechanisms [76]. An earlier study showed the generation of free radicals in dogs after electric shock, which are also very involved in inflammatory processes and programmed cell death [77]. This production of free radicals could be due, in part, to the ultrastructural alterations observed after shock, in particular mitochondrial alterations [78,79].

This cell death is macroscopic and visible. Numerous histopathological studies have found large areas of fibrosis in front of the defibrillation electrodes, especially after multiple electric shocks [80,81,82]. Chronic fibrotic invasion could have an arrhythmogenic effect, with a substrate for localized reentries, and could also promote the inefficiency of defibrillation by raising the high-voltage impedance at the electrode-tissue interface.

### 4.4. Myocardial Dysfunction

The mechanisms of post-shock ventricular stunning are poorly understood and probably numerous. Membrane electroporation due to the electric field also reaches the sarcolemma of the sarcoplasmic reticulum, decreasing its calcium reuptake and storage capacities [83,84]. This direct and immediate effect could explain a transient decrease in myocardial contractility. In addition, cell death leads to the rapid release of large quantities of cations (Mg^++^, K^+^) into the extracellular environment, with consequences for cardiac inotropism in addition to proarrhythmic effects [85].

Myocardial ischemia could also be associated with this phenomenon, although the subject remains debated [86]. Cases of post-shock vasospasm have been described [87]. An episode of ventricular fibrillation rapidly leads to coronary hypoperfusion! In all cases, post-shock contractile dysfunction is more often observed in the hypo-perfused heart [88]. The release of free radicals, directly by ultrastructural alteration, and indirectly by ischemia-reperfusion, could be involved in the dysfunction of the sarcoplasmic reticulum and thus of the contractile function [80,89].

The role of cytosolic calcium overload of the myocyte, through increased membrane permeability and sarcoplasmic reticulum dysfunction, is major, especially after a prolonged episode of ventricular fibrillation [90]. Indeed, administration of verapamil, a calcium channel blocker, attenuates shock-induced myocardial damage in a canine model [91]. Calcium overload would thus promote the reinduction of post-shock ventricular fibrillation, the inefficiency of defibrillation, and ventricular systolic dysfunction in a final fatal vicious circle [92].

As can be seen, the mechanisms involved in defibrillation and their deleterious effects on myocardial tissue are multiple and, above all, poorly understood.

## 5. New Insights from Proteomics

### 5.1. Main Principles

The proteome corresponds to all the proteins of an organism, expressed at a given time and under given conditions. Proteomics will try to identify, in a global way, all the proteins of a cell culture, a tissue, or a biological fluid, and measure their quantity. This is a field of research that has exploded since the beginning of the 2000s, because the identification of the proteome, the final expression of the genome, is an analysis that is much closer to the phenotype than is sequencing, for example.

Proteomic analysis is based on 3 biotechnological tools: mass spectrometry, public databases, and bioinformatics.

Mass spectrometry is the cornerstone of proteomics. It is a very old physical analysis technique, developed by the British Joseph John Thomson, the famous discoverer of the electron. It allows the separation of charged molecules in the gas phase according to their mass/charge ratio (*m*/*z*). A mass spectrometer is composed of four main parts: a sample introduction system; an ionization source, in order to vaporize and ionize the molecules; the analyzer, which separates the ions according to their *m*/*z*; and the detector, which transforms the ions into an electric current that it amplifies for computer processing.

MALDI-TOF mass spectrometry is a “soft” method that uses a matrix-assisted laser desorption ionization (MALDI) source, which minimizes protein degradation. It uses a metal plate receiving a co-crystallized mixture of the matrix and the sample to be analyzed. It is coupled with a time-of-flight (TOF) analyzer, in which the ions are accelerated in an electric field: the ions of the same charge undergo the same acceleration (at constant voltage), but their speed depends on the *m*/*z* ratio, so that the time (of flight) taken by the molecule to reach a detector at a given distance makes it possible to deduce the *m*/*z*.

The “bottom-up” approach is the most used in proteomics. Proteins are first digested by an enzyme (most often trypsin), which results in a mixture of peptides. These peptides are then separated by liquid chromatography before being analyzed by mass spectrometry. The peptides are first identified on the mass spectrum and then fragmented one by one to further improve their identification (tandem-mass spectroscopy MS/MS). The identification of the peptides is done with a specific software, allowing the identification of the original protein(s). The “top-down” approach consists of analyzing a mixture of intact proteins, without pre-digestion [93]. The protein mixture is separated by liquid chromatography, the proteins are then ionized, and the protein to be studied will be isolated and fragmented in the analyzer. The characteristic *m*/*z* of the protein ion and its daughter ions will be used to identify the protein. The advantages of the top-down strategy are its ability to detect degradation products, sequence variants, and combinations of modifications.

### 5.2. Shock-Related Myocardial Injury in Proteomics

**Figure 3:**
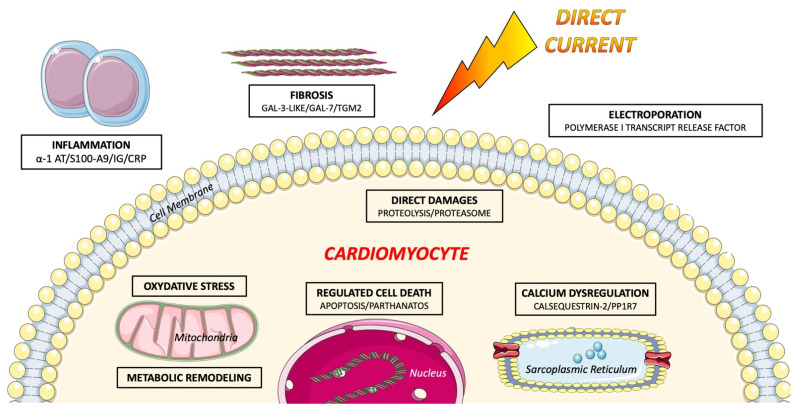
Mechanisms of shock-related myocardial injury identified through a new proteomics approach [94]. Direct current induces electroporation of cardiolemma and direct cellular damages, but also promote regulated cell death, metabolic remodeling, oxidative stress, and calcium dysregulation. Proinflammatory and profibrotic pathways have also been identified.

We recently aimed to highlight the pathophysiological mechanisms involved in the acute phase of electric shocks at the cellular level in an experimental large animal model by utilizing proteomic analysis [94] (Figure 3).

#### 5.2.1. Cytolysis

Mechanisms of cellular destruction were found to be diverse. Enzymatic proteolytic activity was increased following electrical shocks, as suggested by the abundance of proteasome constituents, ubiquitin family proteins, and a decrease in heat shock proteins due to the chaperone effect. Non-enzymatic cell degradation associated with extreme physical conditions may also be involved. Indeed, RAD23 was more abundant after electrical shock and plays a role in the nucleotide excision/repair pathway, especially in the recognition of thermic DNA damages [76]. ATP-dependent (S)-NAD(P)H-hydrate dehydratase was also increased and is known to be transcripted after osmotic or heat stress conditions, in order to convert the abnormal metabolite NAD(P)HX to NAD(P)H [72]. Polymerase I and transcript release factor (PTRF1), a crucial component of the membrane repair machinery, was increased after electrical shocks, suggesting that electroporation-related injury may extend well beyond high-gradient areas [74,95]. Beyond direct myocardial injury, regulated cell death may also play a major role in cardiac dysfunction following electrical shocks. Decrease in inositol monophosphatase, and increase in markers of mitochondrial permeability, transition-driven necrosis, intrinsic apoptosis (VDAC1, VDAC2, HINT2), or parthanatos (poly [ADP-ribose] polymerase 6 isoform X2, AIFM1) were observed [39,42]. Calreticulin may also be implied [82], concordant with indirect evidence of apoptosis in humans [96].

#### 5.2.2. Metabolic Remodeling and Oxidative Stress

Increase of glycolytic pathway was associated with a decrease in upstream keystep of fatty acid metabolism (ACADVL) and gluconeogenesis. This switch from fatty acid β-oxidation to glycolysis is involved in HF [97]. This deep modification of energy metabolism induced by electrical shocks and such metabolic shifts might participate to cardiac dysfunction [98]. Decrease in hemoglobin and myoglobin after electrical shock may also lead to a limitation of oxygene. The abundance of filamin-A (FLNA), involved in mitochondrial hyperfission, induced by hypoxia following myocardial infarction, due to its interaction with Dynamin-related protein 1, may contribute to the mitochondrial ultrastructural alterations in this region already described in dogs, and thus metabolic modifications [78,79]. Aldehyde oxidase production also shows a generation of reactive oxygen species (ROS) such as hydrogen peroxide. Increase in several proteins involved in detoxication of oxygen/nitrogen reactive species suggest an adaptive response to increased oxidative stress. This is concordant with some available data suggesting free-radical generation in dogs following transthoracic shocks [84]. Finally, an increase in peroxisome proliferator-activated receptor γ coactivator 1 (PGC-1) and estrogen-related receptor (ERR)-induced regulator in muscle protein 1 (Perm1), may participate in the regulation of muscle-specific transcriptional programs, such as mitochondrial biogenesis and oxidative metabolism, and adaptation of mitochondrial energy transduction pathways and oxidative metabolism [99].

#### 5.2.3. Calcium Regulation

Interestingly, the cardiomyocyte calcium cycle, a major component of excitation-contraction coupling, is drastically dysregulated following electrical discharges, which may favour both heart failure and ventricular arrhythmias [100]. The decrease in calsequestrin-2 (CASQ2) is known to be associated with a shorter calcium-release phase, but an accelerated restitution of calcium-release sites, with subsequent proarrhythmic calcium concentration oscillations triggering delayed after depolarizations, and subsequent ventricular arrhythmias [101]. Excessive diastolic calcium release may also play a role in the development of acute heart failure [102]. Histidine rich calcium binding protein HRC overexpression may also lead to a depressed contractility [103]. Protein phosphatase 1 (PP1) is also central in SERCA2a regulatome as it dephosphorylates phospholamban (PLN) [104]. An increase of PP1R7 may decrease PP1 activity, dephosphorylated PLN concentration, and thus increase SERCA2a activity and intrasarcoplasmic calcium handling.

#### 5.2.4. Inflammation and Fibrosis

Serpin peptidase inhibitor, clade A, member 1 (also called alpha-1 antitrypsin) and protein S100-A9 increase following electrical shocks, along with immunoglobulins and CRP, suggesting an associated inflammatory response following electrical shocks in a large surrounding area, which may contribute to cardiac dysfunction. The acute increase in galectin 3-like galectin-7 and transglutaminase-2 (TGM2) we observed following electrical shocks might lead to local progressive fibrotic invasion.

## 6. Conclusions

Although electric shocks have been saving many lives every day for more than 50 years, their use should never be trivial. ICDs are sometimes useless, and indications for implantation in the future will probably have to be based on a new stratification of rhythmic risk, particularly on the analysis of the ventricular fibrotic substrate, the truly arrhythmogenic substrate. Once properly implanted, simple programming with a strategy to reduce the quantity of electric shocks delivered, most often unnecessarily, is essential, and has been shown to have a direct beneficial effect on the total mortality of patients implanted with an ICD, particularly in the fragile heart failure population. Indeed, electric shocks immediately cause significant cellular destruction in the ventricular myocardium, major abnormalities in calcium regulation, oxidative stress, and stimulation of pro-inflammatory and pro-fibrotic phenomena, which promote contractile dysfunction and the paradoxical reinduction of ventricular arrhythmias.

## 7. Outlook

Regarding the reduction of therapies, a gradual change in the programming habits of ICDs is under way and must continue, in compliance with recommendations. Perhaps the next version of the expert recommendations will hopefully be even more permissive.

The method of defibrillation itself may simply need to be modified, and high energies abandoned. New extracardiac defibrillation technologies may further reduce the deleterious effects of electric shocks—as the energy is not delivered directly into the heart chambers—and thus may improve shock efficiency [105]. Subcutaneous ICD-S-ICD has been developed to overcome systemic infections and failure of transvenous leads, the most frightening complications of conventional ICDs. However, this technology still delivers high-energy shocks at 80 J (40 J for conventional ICDs), and has no anti-bradycardia nor anti-tachycardia pacing capability. Although some preliminary studies already suggest that extra-cardiac defibrillation might reduce myocardial injury, further studies are needed to explore the deleterious effects of these alternative technologies [106].

Finally, new approaches such as the use of “shock bursts” (the successive delivery of several low-energy shocks) or, in a more distant future, optogenetics (the use of light to modify transmembrane potentials) may hopefully put a definitive end to the toxic effects of electric shocks [107,108].

## Data Availability

Not applicable.
